# *Sasa borealis* Stem Extract Attenuates Hepatic Steatosis in High-Fat Diet-induced Obese Rats

**DOI:** 10.3390/nu6062179

**Published:** 2014-06-05

**Authors:** Yuno Song, Soo-Jung Lee, Sun-Hee Jang, Ji Hee Ha, Young Min Song, Yeoung-Gyu Ko, Hong-Duck Kim, Wongi Min, Suk Nam Kang, Jae-Hyeon Cho

**Affiliations:** 1 Institute of Agriculture and Life Science, College of Veterinary Medicine, Gyeongsang National University, Jinju 660-701, Korea; E-Mails: yunosong0805@gmail.com (Y.S.); sunhee5321@naver.com (S.-H.J.); wongimin@gnu.ac.kr (W.M.); 2 Department of Foods and Nutrition, Gyeongsang National University, Jinju 660-701, Korea; E-Mail: bodry96@hanmail.net; 3 Department of Animal Science & Biotechnology, Gyeongnam National University of Science and Technology, Jinju 660-758, Korea; E-Mails: hee71111@nate.com (J.H.H.); pigsong@gntech.ac.kr (Y.M.S.); 4 Animal Genetic Resources Station, National Institute of Animal Science, RDA, Namwon 590-832, Korea; E-Mail: kog4556@korea.kr; 5 Department of Environmental Health Science, New York Medical College, Valhalla, NY 10595, USA; E-Mail: HongDuck_Kim@nymc.edu; 6 Department of Bioindustry, Daegu University, Gyungsan 712-714, Korea

**Keywords:** high-fat diet, *Sasa borealis* stem, hepatic steatosis, hepatic gene expression

## Abstract

The aim of the current study is to examine the improving effect of *Sasa borealis* stem (SBS) extract extracts on high-fat diet (HFD)-induced hepatic steatosis in rats. To determine the hepatoprotective effect of SBS, we fed rats a normal regular diet (ND), HFD, and HFD supplemented with 150 mg/kg body weight (BW) SBS extracts for five weeks. We found that the body weight and liver weight of rats in the HFD + SBS group were significantly lower than those in the HFD group. Significantly lower serum total cholesterol (TC) and triglyceride (TG) concentrations were observed in the SBS-supplemented group compared with the HFD group. We also found that the HFD supplemented with SBS group showed dramatically reduced hepatic lipid accumulation compared to the HFD alone group, and administration of SBS resulted in dramatic suppression of TG, TC in the HFD-induced fatty liver. In liver gene expression within the SBS treated group, PPARα was significantly increased and SREBP-1c was significantly suppressed. SBS induced a significant decrease in the hepatic mRNA levels of PPARγ, FAS, ACC1, and DGAT2. In conclusion, SBS improved cholesterol metabolism, decreased lipogenesis, and increased lipid oxidation in HFD-induced hepatic steatosis in rats, implying a potential application in treatment of non-alcoholic fatty liver disease.

## 1. Introduction

Obesity-related nonalcoholic fatty liver disease (NAFLD), one of the most common liver diseases, is significantly associated with metabolic syndrome, including obesity, dyslipidemia, and insulin resistance [1,2]. A hypercaloric dietary habit easily results in increased body weight, serum lipids, and hepatic lipid accumulation. Increased liver lipid accumulation causes lipid peroxidation, leading to further advancement of liver damage. Accumulation of triglycerides, termed as hepatic steatosis, which is characterized by fibrosis and necroinflammation, and can progress to cirrhosis and terminal liver failure, has been proposed as an indication of more severe liver disease [3]. Of particular importance, hepatic steatosis is always coupled with other diseases, *i.e.*, obesity, diabetes, and hyperlipidemia [4]. Therefore, the clinical implications of hepatic steatosis are due mainly to its potential to cause chronic inflammation and then progress to cirrhosis and liver failure.

One of the major causes of lipid accumulation in NAFLD is the inability of the liver to regulate changes in lipogenesis in the transition from fasted to fed state [5]. Several studies have suggested that hepatic lipogenesis is increased in hepatic steatosis, which may result from either increased triglyceride synthesis, or decreased fatty acid oxidation through production of malony-CoA, both leading to increased triglyceride content in the liver [6]. Excess fat accumulation ultimately leads to development of hepatic steatosis and worsening hepatic insulin resistance via a network of transcription factors [7], which regulate hepatic lipogenesis and fatty acid oxidation, including sterol regulatory element-binding protein-1c (SREBP-1c), liver X receptor, and peroxisome proliferator receptors (PPARs). In addition, normalizing serum lipids is also known to be a way to hinder the occurrence of hepatic steatosis. The potential lipid lowering effect might be mediated by down-regulation of various lipogenic enzyme activities. Enzymes of the lipogenic pathway that are transcriptionally regulated include acetyl-CoA carboxylase (ACC), fatty-acid synthase (FAS), diacylglycerol acyltransferase2 (DGAT2), and stearoyl-CoA desaturase (SCD-1). 

There has been a substantial effort toward understanding the mechanisms underlying NAFLD induced metabolic disorders and numerous studies have been conducted in the search for natural active products, particularly potential sources of antioxidant [8]. Bamboo has been used for medicinal purposes for centuries in Korea and other Asian countries, and its efficacy has been recorded in the materia medica, DongEuiBoGam in Korea [9,10]. Recent scientific research has demonstrated the health benefits of Bamboo leaves [10,11], culm [12], shoot [13], and shavings [14].

The medicinal effects of *Sasa borealis*, a species of Bamboo, are predominantly anti-diabetic through enhancement of insulin secretion [15], hypoglycemic and hypolipidemic effects [11], anti-obesity [16] and/or anti-oxidant effects [17]. In addition, clinical uses of Bamboo for treatment of hypertension, arteriosclerosis, cardiovascular disease, and cancer have been described [18]. 

Currently, no studies investigating the effects of dietary supplementation with *Sasa borealis* stem (SBS) extracts on fat metabolism in a high-fat diet (HFD)-induced NAFLD model have been reported. In the current study, we investigated the effects of SBS on lipid levels and cholesterol levels in the liver and serum of rats fed a HFD, and on expression of genes involved in fatty acid synthesis and oxidation in the liver.

## 2. Experimental Section

### 2.1. Preparation of Sasa borealis Stem (SBS) Extracts

Briefly, the stems of *Sasa borealis* were collected during the autumn season in Gyeongnam Province, Korea. The coarse powder of SBS was obtained after comminution and filtration (20–40 mesh) and 20 g powder were ground in an 80% (v/v) methanol solution using a mixer, followed by extraction of the samples for three days with vigorous shaking. The filtrate was then isolated by membrane filtration for removal of macro- and micro-molecular components, such as polysaccharides and minerals. The extraction yield from dry weight of SBS was 19.6%. The methanol extracts of the SBS were concentrated using rotary-vacuum evaporation at 50 °C and then freeze-dried.

### 2.2. Measurement of Total Phenolic Content Using the Folin-Ciocalteu Assay

The total phenolic content of the SBS was determined using a spectrophotometer according to the Folin-Ciocalteu colorimetric method [19]. Because gallic acid is one of the polyphenol compounds found in SBS, the total phenolic content of methanol extract of SBS was expressed as mg gallic acid (Sigma-Aldrich, USA) equivalents (GAE)/g. 

### 2.3. Measurement of Total Flavonoids

The total flavonoid content was determined as previously described [20] with slight modifications. Briefly, 0.25 mL of SBS extracts (100 µg/mL) was added to a tube containing 1 mL of double-distilled water. Next, 0.075 mL of 5% NaNO_2_, 0.075 mL of 10% AlCl_3_, and 0.5 mL of 1 M NaOH were added sequentially at 0, 5, and 6 min. Finally, the volume of the reacting solution was adjusted to 2.5 mL with double-distilled water. The solution had an absorbance of 410 nm, which was detected using an Ultrospec 2100 Pro Spectrophotometer (Section 3.3). The results were expressed in mg quercetin equivalents (QE)/g.

### 2.4. Measurement of Free Radical Scavenging Activity Using the 2,2-Diphenyl-1-picrylhydrazyl (DPPH) Assay

The free radical scavenging activity of SBS (100 µg/mL in DW) was measured using the method of Brand-Williams [21] with some modification. l-Ascorbic acid was used as a positive control. The inhibition percentage was calculated from the following equation:

Inhibition% = [(absorbance of control-absorbance of sample)/absorbance of control] × 100
(1)

The absorbance was measured using a spectrophotometer (Ultrospec 2100 pro; Amersham Pharmacia Biotech Co., Piscataway, NJ, USA). 

### 2.5. Measurement of Hydroxyl (OH−) Radical Scavenging Activity

Scavenging activity of SBS extracts on the hydroxyl radical (OH**^−^**) was measured using the deoxyribose method [22] with a slight modification. The deoxyribose assay was performed in 10 mM phosphate buffer (pH 7.4) containing 2.5 mM deoxyribose, 1.5 mM H_2_O_2_, 100 μM FeCl_3_, 104 μM EDTA, and the extracts (1 mg/mL). The reaction was started by addition of ascorbic acid to the final concentration of 100 μM. The reaction mixture was incubated at 37 °C for 1 h in a water-bath and after incubation, the color was developed by addition of 0.5% thiobarbituric acid followed by ice-cold 2.8% trichloroacetic acid in 25 mM NaOH and heated at 80 °C for 30 min. The extracts (*A*2) were cooled on ice and the absorbance was measured at 532 nm. The reaction mixture without the test sample was used as a control (*A*1). The hydroxyl radical scavenging activity (HRSA) was calculated using the following equation:

HRSA% = (*A*1 − *A*2/*A*1) × 100 (2)

The inhibition curve was plotted for four experiments and was expressed as the % of the mean inhibition ±SD. 

### 2.6. Measurement of ABTS Radical Scavenging Activity

ABTS radical scavenging activity of SBS extracts and fractions was measured using the ABTS cation decolorization assay, as previously described [23], with some modifications. The ABTS radical cation (ABTS^•+^) was produced by reaction of 7 mM stock solution of ABTS with 2.45 mM potassium persulfate and allowing the mixture to stand in the dark at room temperature for 12 h before use. The ABTS^•+^ solution was diluted with methanol to give an absorbance of 0.7 ± 0.01 at 734 nm. Plant extracts and fractions (1 mL) were allowed to react with 2 mL of the ABTS^•+^ solution and the absorbance was measured at 734 nm after 1 min. Trolox was used as a reference compound. The results were expressed as Trolox equivalent antioxidant capacity (TEAC) values and calculated as mean value ± SD (*n* = 4). 

### 2.7. High-Performance Liquid Chromatography (HPLC) Analysis of SBS Compounds

Analysis of the compounds in the extract was performed using an Agilent 1100 series HPLC unit, equipped with a DAD. Samples were separated on a Nucleosil 100-5 C-18 column (250 mm × 4.0 mm, i.d., 5 μm particle size) which was protected by a 10 mm guard column with a gradient elution system. The mobile phase consisted of two solvents: solvent A was a mixture of water/formic acid (pH 3.29), and solvent B was 100% acetonitril/formic acid (pH 3.29). Gradient elution was performed as follows: initially, 7.0% of solvent B, followed by 0% to 15% B in 25 min, 30% B at 35 min, 40% B at 50 min, 100% B at 45 min, and 100% B at 55 min. The flow rate was 1.0 mL/min, and the column temperature was 30 °C. Used phenolic or flavonoid standard were performed by comparing retention times. The injections of sample and standard were performed in triplicate.

### 2.8. Animals and Diets

Four-week-old male Sprague-Dawley (Central Lab. Animal Inc.) rats were purchased from Central Lab. Animal Inc. (Seoul, Korea). Rats were acclimatized to the experimental facility for one week prior to the start of the study. The rats were divided into three groups of 10 and housed individually in polycarbonate cages in a room maintained at 22 °C and 55% relative humidity. The room was exposed to alternating 12 h periods of light and dark. All of the rats were allowed free access to food and water for five weeks. Food intake was measured daily, and the rats were weighed twice per week. After one week of acclimatization, rats were randomly divided into three groups: a normal diet group (ND, *n* = 10), a high-fat diet group (HFD, *n* = 10), and a SBS group (HFD + SBS 150 mg/kg BW, *n* = 10). Rats in the ND group were fed a normal diet (#55VXT0038, Samyang Co, Korea). Obese rats were generated by feeding rats a high-fat diet, and rats in the HFD groups were fed a HFD based on a commercial diet (rodent diet with 60% kcal fat, Research Diet, Korea). The composition of the diets and energy densities were presented in Table 1. The study protocol was approved by the Animal Care and Use Committee of Gyeongsang National University (Approval Number: GNU-130525-R0042).

**Table 1:** Ingredient composition of the experimental diet.

Ingredient (milligram)	HFD	HFD + SBS (mg/kg/day)
Casein	200	200
l-Cystine	3	3
Maltodextrin 10	125	125
Sucrose	68.8	68.8
Cellulose	50	50
Soybean Oil	25	25
Lard	245	245
Mineral Mix S10026	10	10
DiCalcium Phosphate	13	13
Calcium Carbonate	5.5	5.5
Potassuium Citrate	16.5	16.5
Vitamin Mix V10001	10	10
Choline Bitartrate	2	2
Protein (milligram%)	26.2	26.2
Carbohydrate (milligram%)	26.3	26.3
Fat (milligram%)	34.9	34.9
SBS extracts	-	150

### 2.9. Biochemical Analysis

Biochemical analysis was performed using commercial kits. Blood was placed in tubes containing EDTA_2_Na, and serum was obtained by centrifuging the blood at 3000× *g* 10 min at 4 °C. Serum levels of aspartate aminotransferase (AST) and alanine aminotransferase (ALT) were detected using a Dry-chem Chemistry analyzer (Fujifilm, Japan). Excessive accumulation of triglyceride (TG) in the liver is the hallmark of NAFLD. Serum TG levels were assayed enzymatically using commercial kits (Asan phams, Co., Korea). For determination of hepatic TG content, 250 mg of liver was homogenized in 4 mL of chloroform/methanol (2:1, v/v), and 1 mL of 50 mM NaCl was added to each sample. The samples were then centrifuged, and the organic layer was removed and dried. The resulting pellet was dissolved in phosphate-buffered saline containing 1% Triton X-100, and the triglyceride content was determined using a commercially available enzymatic reagent kit (Asan phams, Co., Korea). The concentrations of total-cholesterol (TC) and high-density lipoprotein (HDL)-cholesterol were assayed enzymatically using commercial kits (Asan phams, Co., Korea).

### 2.10. Histopathological Examinations

Liver samples obtained at 12 h after the last administration of SBS extracts were fixed in 4% phosphate-buffered 4% Paraformaldehyde, then processed routinely, embedded in paraffin, and sectioned to 5 µm thickness. The sections were deparaffinized, rehydrated using standard techniques, stained with hematoxylin and eosin (H and E), and examined by light microscopy. 

### 2.11. Gene Expression Analysis

Total RNA was isolated from liver tissues from the different rat groups using Trizol reagent (Invitrogen, CA, USA). One microgram of total RNA was subjected to first strand cDNA synthesis using oligo (deoxythymidine) primers and Superscript II reverse transcriptase (Invitrogen, CA, USA). The target cDNA was amplified using the following sense and antisense primers: sense 5′-GGA GCC ATGGATTGCACATT-3′ and antisense 5′-AGGAAGGCTTCCAGAGAGGA-3′ for SREBP-1c; sense 5′-AAGGCTATCCCAGGCTTTGC-3′ and antisense 5′-CGTCTGACTCGGTCTTCTTG-3′ for PPARα; sense 5′-TTTTCAAGGGTGCCAGTTTC-3′ and antisense 5′-AATCCTTGGCCCTCTGAGAT-3′ for PPARγ; sense 5′-TGCTAGAGGCCCTGCTACCAC-3′ and antisense 5′-TGTGCACAGACACCTTCCCATC-3′ for FAS; sense 5′-AGGAAGATGGTGTCC CGCTCTG-3′ and antisense 5′-GGGGAGATGTGCTGGGTCAT-3′ for ACC; sense 5′-TACAAGCAGGTGATCTTTGAGG-3′ and antisense 5′-GGGCGAAACCAATATACTTCTG-3′ for DGTA2. Control detection of β-actin was performed using sense 5′-AGGTCATCACTATCGGCAAT-3′ and antisense 5′-ACTCATCGTACTCCTGCTTG-3′ primers. PCR products were separated by electrophoresis on 1.5% agarose gel for 30 min at 100 V. Gels were stained with 1 mg/mL ethidium bromide visualized by UV light using BIO-RAD Gel Doc image analysis software (BIO-RAD Laboratories Inc., CA, USA). All PCR products measured were normalized to the amount of β-actin cDNA in each sample. The mPNA levels are expressed as a ratio relative to β-actin mRNA. 

### 2.12. Statistical Analysis

Data are expressed as mean ± SD. Significant differences among the treatment means were determined using ANOVA followed by Tukey’s multiple comparisons test. *p* < 0.05 was considered statistically significant.

## 3. Results

### 3.1. Total Phenol Content (TPC) and Total Flavonoid Content (TFC) of SBS Extracts

Data from analysis of total phenolic and flavonoid contents in the methanol extract from SBS are shown in Table 2. The phenolic contents of the extracts are shown as (+)-catechin equivalents (CQ) and flavonoids as quercetin equivalents (QE). The total phenolic content was 430.3 mg CE/g dry weight and the total flavonoid content of SBS extracts was 127.5 mg QE/g dry weight, respectively (Table 2).

**Table 2:** Antioxidant capacities, total phenolic and flavonoids content of *Sasa borealis* stem (SBS) extracts.

	TPC (mg Gallic acid/g extract)	TFC (mg Quercetin/g extract)	IC50 (µg/mL)		ABTS assay TEAC
			DPPH scavenging assay	HRSA scavenging assay	
SBS	430.0 ± 40.2	127.5 ± 12.0	43.7 ± 2.3	450.5 ± 28.5	0.6 ± 0.05

### 3.2. Antioxidant Activity of SBS Extracts

The antioxidant activities of SBS extracts were determined using the DPPH, ABTS, and HRSA assays. Data on DPPH radical scavenging activity of SBS are shown in Table 2. The results were expressed as IC_50_, which indicates the antioxidant concentration necessary for scavenging the initial DPPH concentration by 50%. The SBS extract also inhibited hydroxyl radical generation and was found to possess strong antioxidant activity in hydroxyl radical scavenging activity. Trolox equivalent antioxidant capacity (TEAC) assay is one of the most commonly employed methods for determining antioxidant capacity. The TEAC assay measures the ability of a compound to scavenge ABTS radicals, and is widely used in screening of antioxidant activity of fruits, vegetables, and plants. The result of antioxidant activity of SBS extracts was expressed as TEAC values, as shown in Table 2. SBS showed high radical scavenging potential. 

TPC, total phenolic content; TPC, total flavonoid content. Data are presented as the mean ± SD (*n* = 4). TPC expressed as milligrams of gallic acid per gram of dry weight. TFC expressed as milligrams of Quercertin equivalent per gram of dry weight. Scavenging of free radicals by SBS extracts according to DPPH, Hydroxyl (OH^−^), and ABTS scavenging assay. DPPH, DPPH radical scavenging activity; ABST, ABST radical scavenging activity; HRSA, hydroxyl radial scavenging activity. Data are presented as mean ± SD (*n* = 4). The antioxidant activity was evaluated as the concentration of tested sample required to scavenge 50% of the DPPH and HRSA. Trolox equivalent antioxidant capacity (TEAC) assay measured the ability of SBS to scavenge ABTS radicals. SBS extracts also contained phloroglucinol, 4-hydroxy benzhydrazide, garlic acid, vanillic acid, caffeic acid, syringic acid, chlorogenic acid, *p*-coumaric acid, *trans*-ferulic acid, sinapic acid, 2-amino-3,4-dimethyl-benzoic acid, protocatechuic acid, and coumarin. Among phenolic compounds, protocatechuic acid, coumarin, and *p*-coumaric acid were the main materials. In addition, SBS extracts also had many flavonoids, such as gallocatechin, epigallocatechin, catechin hydrate, epicatechin, epigallocatechin gallate, rutin hydrate, naringin, quercetin hydrate, myricetin, quercetin dehydrate, luteolin, and kaempferol (Table 3).

**Table 3:** Concentrations of phenolic acids and flavonoids of SBS extracts.

Standards	RT ^1^	λ ^2^	Calibration curve	LOQ ^3^	Compounds
*Phenolic acids*					
Phloroglucinol	7.27	280	*Y* = 397.949*X* + 0.655	50.00	0.60 ± 0.00
4-Hydroxy benzhydrazide derivative	7.57	280	*Y* = 8119.555*X* − 59.083	50.00	0.26 ± 0.00
Gallic acid	8.74	280	*Y* = 18,200.182*X* − 28.003	50.00	0.53 ± 0.00
Vanillic acid	21.91	280	*Y* = 11,026.185*X* + 14.026	50.00	1.44 ± 0.01
Caffeic acid	22.21	280	*Y* = 19,697.774*X* − 13.018	50.00	0.74 ± 0.00
Syringic acid	24.10	280	*Y* = 17,500.224*X* − 1.523	5.00	1.41 ± 0.01
Chlorogenic acid	24.92	280	*Y* = 6240.064*X* − 10.524	50.00	1.61 ± 0.01
*p*-Coumaric acid	32.87	280	*Y* = 23,926.358*X* + 0.631	5.00	10.41 ± 0.05
*trans*-Ferulic acid	34.48	280	*Y* = 16,058.167*X* − 17.063	50.00	3.44 ± 0.02
Sinapic acid	34.91	280	*Y* = 7025.930*X* + 0.785	50.00	2.98 ± 0.01
2-Amino-3,4-dimethyl-benzoicacid	35.30	280	*Y* = 1209.000*X* + 0.000	50.00	6.95 ± 0.04
*p*-Anisic acid	35.40	280	*Y* = 9558.576*X* + 5.493	50.00	-
Protocatechuic acid ethyl ester	36.89	280	*Y* = 8796.340*X* − 2.765	50.00	13.05 ± 0.06
Coumarin	38.27	280	*Y* = 24,055.754*X* + 48.641	4.00	14.91 ± 0.04
DPBA ^4^	39.85	280	*Y* = 2971.415*X* − 7.872	50.00	-
Alizarin	43.86	280	*Y* = 15,428.805*X* + 19.936	1.00	-
Total phenolic acids					62.98 ± 0.08
*Flavonoids*					
Gallocatechin	17.68	280	*Y* = 1331.637*X* + 0.000	50.00	1.59 ± 0.00
Epigallocatechin	18.58	280	*Y* = 96.137*X* − 0.550	50.00	0.73 ± 0.00
Catechin hydrate	23.66	280	*Y* = 3982.083*X* − 6.943	5.00	2.65 ± 0.01
Epicatechin	28.00	280	*Y* = 7641.670*X* − 14.487	50.00	2.47 ± 0.00
Epigallocatechin gallate	29.53	280	*Y* = 6425.894*X* − 6.592	50.00	0.98 ± 0.00
Rutin hydrate	32.93	370	*Y* = 4763.242*X* − 4.752	50.00	0.29 ± 0.01
Catechin gallate	33.77	280	*Y* = 1462.905*X* − 1.970	50.00	-
Naringin	34.14	280	*Y* = 8230.457*X* − 42.997	50.00	2.60 ± 0.02
Quercetin hydrate	37.53	370	*Y* = 7476.858*X* − 6.972	50.00	2.48 ± 0.02
Myricetin	37.41	370	*Y* = 9908.955*X* − 0.383	5.00	0.43 ± 0.00
Morin hydrate	38.48	320	*Y* = 4100.693*X* − 4.129	50.00	-
Quercetin dehydrate	40.19	370	*Y* = 5623.574*X* − 0.729	50.00	1.93 ± 0.00
Luteolin	40.28	370	*Y* = 12,303.249*X* − 8.820	50.00	6.32 ± 0.03
Kaempferol	42.89	370	*Y* = 12,894.258*X* + 38.962	20.00	0.95 ± 0.03
3-Hydroxyflavone	45.75	320	*Y* = 4687.303*X* + 0.191	50.00	-
Total flavonoids					23.42 ± 0.09

Table 3 expressed by the RT, λ, calibration curve, LOQ and detected compounds. ^1^ RT, retention time; ^2^ λ, absorbance (nm); ^3^ LOQ, limit of quantitation; ^4^ DPBA, diphenylboric acid 2-aminoethyl ester. Data are presented as mean ± SD (*n* = 3).

### 3.3. Effect of SBS on Body Weight and Liver Weight in HFD-Fed Rats

Body weight gain and food intake of animals fed the experimental diets are shown in Table 4. No significant difference in food intake was observed among the groups during the experimental diet period. Consumption of HFD (Table 1) for five weeks resulted in significantly increased body weight compared to the normal diet (ND). In contrast, rats fed a HFD supplemented with SBS extracts had 30.5% lower body weights than rats fed a HFD alone. The mean liver weight in the HFD group was significantly increased compared to that of the ND group. However, the increases in mean liver weight in the HFD + SBS group were smaller than those in the HFD group (Table 4). Although administration of HFD was found to result in significant elevation of the liver weight of the HFD group compared to the ND group, treatment with SBS was found to result in strong attenuation of liver weight.

**Table 4:** Effects of SBS on body and liver weights in high-fat diet (HFD)-fed rats.

	ND	HFD	HFD + SBS
Food intake (g/day)	12.03 ± 1.1	11.04 ± 1.6	11.44 ± 1.5
Body weight			
Initial weight (g)	136.2 ± 4.3	135.5 ± 2.7	134.2 ± 3.7
Final weight (g)	338.7 ± 16.8	447.3 ± 20.5 *	388.5 ± 15.5 ^#^
Weight gain (g/5 weeks)	202.5 ± 9.3	311.8 ± 16.3 *	254.3 ± 10.7 ^#^
Liver weight (g/5 weeks)	2.5 ± 0.2	3.8 ± 0.3 *	3.2 ± 0.3 ^#^

Rats fed a HFD were treated orally with SBS extracts at a dose of 150 mg/kg body weight. ND: normal diet group, HFD: High-fat diet group, HFD + SBS: High-fat diet plus SBS (150 mg/kg BW) group. Body weight was measured twice per week. The weight of the liver was calculated by dividing the liver tissue weight by body weight (liver tissue/body weight × 100). The values are expressed as the mean ± SD (*n* = 10). * *p* < 0.01 compared to ND. ^#^ *p* < 0.05 compared to HFD.

### 3.4. Effect of SBS on Serum Total Cholesterols and Triglyceride Levels

**Figure 1:**
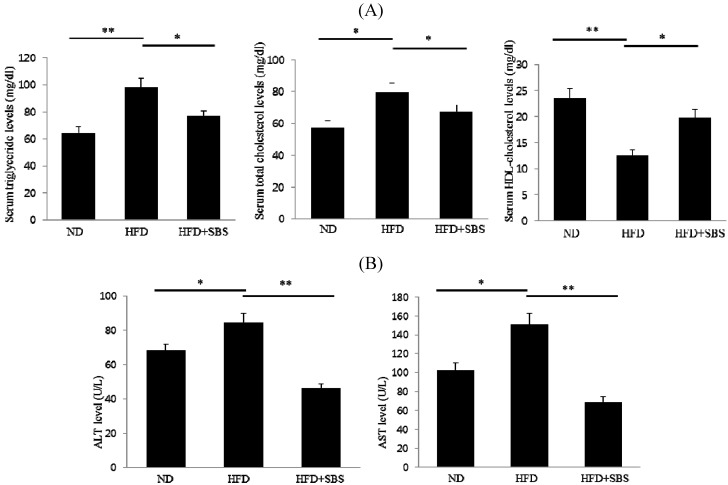
Effect of SBS on serum lipid contents in HFD-fed rats. (**A**) Serum TG and TC levels in rats fed a normal diet (ND), high-fat diet (HFD), and high-fat diet supplemented with SBS. Serum TG and TC levels were significantly reduced in rats treated with HFD + SBS compared to HFD. The values are expressed as the mean ± SD. * *p* < 0.05, ** *p* < 0.01. (**B**) Serum levels of AST and ALT of rats fed ND, HFD, and HFD + SBS. The values are expressed as the mean ± SD. * *p* < 0.05, ** *p* < 0.01.

To determine hepatic steatosis-preventing the effect of SBS on HFD-fed rats, the serum lipid levels were measured. The HFD group showed significantly elevated serum TG concentrations compared to the ND group (Figure 1A). Administration of SBS resulted in decreased serum TG by 35% compared with the HFD group (Figure 1A). In addition, we found that the TG levels in the SBS group were close to those in the ND group. The HFD group also showed significantly increased levels of TC relative to the ND group, while SBS caused a marked decrease in serum TC levels when compared to the HFD group. The serum HDL-cholesterol level was significantly increased in the HFD + SBS group, when compared with HFD alone (Figure 1A). For evaluation of liver function, ALT and AST activities were examined between groups. Significantly increased ALT levels were observed in the HFD group compared to the ND group, while treatment with SBS resulted in a marked decrease in the ALT level compared to the HFD group (Figure 1B). AST levels in rats supplemented with SBS were significantly lowered compared to those in the HFD group (Figure 1B).

### 3.5. Effects of SBS on Hepatic Total Cholesterols Levels and Triglyceride Levels

**Figure 2:**
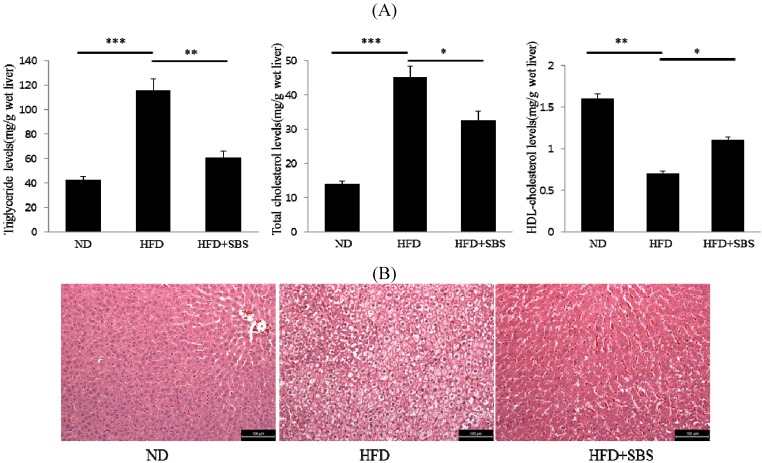
SBS reduces hepatic lipid levels in HFD-induced hepatic steatosis in rats. (**A**) Hepatic TG and TC levels in rats fed a normal diet (ND), high-fat diet (HFD), and high-fat diet supplemented with SBS. Significant decreases in the levels of hepatic triglyceride and total cholesterol were observed in the SBS-treated groups compared with HFD-induced obese rats. Values are expressed as mean ± SD. * *p* < 0.05, ** *p* < 0.01. (**B**) Representative histological section of H & E staining of liver prepared from rats fed ND, HFD, and HFD supplemented with SBS (magnification 200×, scale bar = 100 µm). The major histological change induced by HFD in rat liver was hepatocyte steatosis and ballooning.

To examine the effect of SBS on biochemical changes, we determined the levels of TG and TC in the liver. Administration of a HFD was found to result in significantly increased hepatic TG and TC in the HFD group. The SBS group, treated with SBS, showed markedly lower levels of TG and TC in the liver (Figure 2A). In addition, after a HFD for five weeks, the hepatic HDL-cholesterol levels showed a decrease compared with the ND group. However, the hepatic HDL-cholesterol levels in the SBS group increased by approximately 40% compared with the levels from rats on a HFD (Figure 2A). These results can be attributed to the ability of SBS to effectively suppress accumulation of hepatic TG and TC in HFD-fed rats.

### 3.6. Effects of SBS on Hapatic Steatosis

Next, H and E staining was performed for analysis of the effect of SBS on HFD-induced lipid accumulation in the liver. Lipid accumulation was highly amplified in the HFD group compared to the ND group. Histological examination of livers also revealed significant hepatic steatosis-characterized by swelling of hepatocytes and fat accumulation—in rats fed a HFD as compared with the HFD + SBS groups (Figure 2B). However, the lipid accumulations induced by HFD were significantly reduced in the HFD + SBS group, indicating that SBS is capable of preventing lipid accumulation and hepatic steatosis in HFD-induced fatty liver. 

### 3.7. Effects of SBS on Hepatic mRNA Levels of Lipid-Related Gene Expression

**Figure 3:**
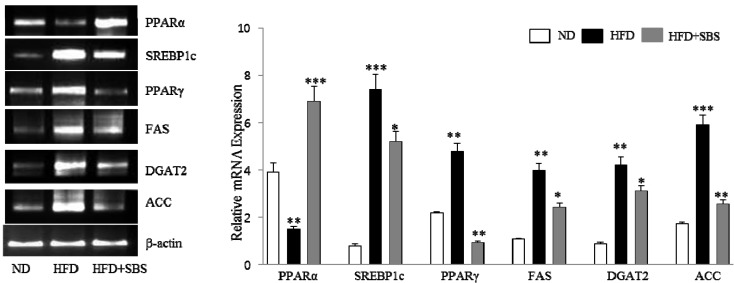
SBS inhibits expression of genes that regulate hepatic lipogenesis and fatty acid oxidation in hepatic steatosis. Total RNA isolated from liver was subjected to RT-PCR, and all of the gene transcripts were normalized using β-actin as a control. All of the experiments were performed in three independent experiments. Mean valves were significantly different from the ND group: * *p* < 0.05; ** *p* < 0.01; and *** *p* < 0.001. ND, rats fed a normal diet; HFD, rats fed a high-fat diet; HFD + SBS, rats fed a high-fat diet plus SBS (150 mg/kg BW); SREBP-1c, sterol regulatory element-binding protein-1c; PPARα, peroxisome proliferator receptors-alpha; PPARγ, peroxisome proliferator receptors-gamma; FAS, fatty-acid synthase; ACC, acetyl-CoA carboxylase; DGTA2, diacylglycerol acyltransferase 2.

For evaluation of the molecular events underlying the effects of SBS, we analyzed the expression of genes involved in lipid homeostasis in the liver. We assessed hepatic levels of lipogenesis-related genes (SREBP-1c, PPARγ, and FAS), as well as fatty acid metabolism-related genes (PPARα, ACC, and DGAT2) in HFD-fed rats. According to our results, significantly lower gene expression levels of SREBP-1c, PPARγ, and FAS, which promote synthesis of *de novo* monounsaturated fatty acid, were observed in the SBS-treated group than in the HFD group (Figure 3). Of these genes, expression of SREBP-1c and PPARγ showed the most significant reduction in the SBS group, compared to the HFD group (Figure 3). Hepatic mRNAs for PPARα and ACC, the genes involved in fatty acid β-oxidation, were significantly decreased by BS administration when compared to the HFD group. In addition, SBS supplementation induced a significant decrease in the mRNA level of DGAT2 compared to the HFD group (Figure 3). Together, these results demonstrated that SBS contributed to inhibition of fatty acid synthesis, while activating fatty acid β-oxidation in livers of HFD-fed rats. 

## 4. Discussion

Despite the rapidly growing recognition of hepatic steatosis over the past decade, therapy directed at treatment or prevention of the disease remains. Given the high prevalence of obesity in patients with hepatic steatosis, prevention of hepatic fat accumulation through weight reduction remains the cornerstone of treatment of hepatic steatosis [24]. In the current study, we investigated the anti-hepatic steatosis activity of SBS extract in HFD-fed rats. Our results demonstrated that, compared to the ND group, noticeably greater lipid accumulation in liver tissue and dramatically increased body and liver weights were observed in the HFD group. However, SBS supplementation in rats fed a HFD was effective in decreasing liver triglyceride and total cholesterol, and resulted in marked lowering of liver weight increases compared to the HFD group. 

High fat diets cause weight gain, fat accumulation, and increased fat levels in the liver and serum. Fat accumulation in the liver increases the risks of NAFLD and non-alcoholic steatohepatitis (NASH), which cause hypercholesterolemia and cardiovascular disease [25]. Regarding the changes in lipid metabolism caused by fat accumulation, the aminotransferase activity of the liver is altered. Measurement of liver damage caused by fat accumulation in the liver is important for diagnosis of NAFLD. Therefore, AST and ALT were investigated as markers of liver damage. In the current study, levels of aspartate aminotransferase (AST) and alanine aminotransferase (ALT) showed a more significant increase in rats fed a HFD, compared to rats fed a ND. However, SBS supplementation resulted in significantly decreased levels of ALT and AST, demonstrating the robust hepatoprotective effects of SBS against HFD-induced liver damage. 

Obesity causes altered function of adipocytes, which leads to expanded adipocyte mass and increased release of FFAs in the blood, which increases the amount of TG stored in the liver. Excess storage of TG in the liver results in significant and more abundant lipid accumulation, resulting in fatty liver [26,27]. In this study, rats in the HFD group showed a large number of lipid droplets and increased TG and cholesterol concentration in the liver and serum, confirming development of hepatic steatosis in the animal model. Administration of SBS to rats fed a HFD resulted in significantly decreased body weight as well as liver tissue weight. Treatment with SBS also resulted in a significant decrease in the hepatic and serum TG concentrations in HFD-induced hepatic steatosis in rats. In addition, our data from histopathological examination of livers from the rats showed a significant increase in the number and size of fatty hepatocytes upon HFD administration but returned to normal levels in rats that were administered SBS. These results demonstrated that the HFD-induced hepatic pathological changes were significantly inhibited in SBS-fed rats. These results clearly demonstrated that treatment with SBS resulted in effective improvement of hepatic steatosis induced by HFD.

The chemical compound cholesterol is a combination of lipid and steroid and is produced naturally by the body. Approximately 80% of the body’s cholesterol is produced by and stored in the liver [28]. The liver is able to regulate cholesterol levels in the bloodstream and can secrete cholesterol if it is needed by the body. In the current study, we showed that administration of a HFD resulted in significantly increased levels of TC and decreased levels of HDL-cholesterol in serum and liver. However, administration of SBS resulted in decreased levels of serum and liver TC compared to the HFD group. In addition, SBS-fed rats showed liver HDL-cholesterol levels similar to those of the ND group. 

Next, to explore the possible mechanism of SBS in decreasing accumulation of liver lipids, we investigated the expression levels of several genes related to fatty acid transport, and lipid metabolism, including lipogenesis and β-oxidation. Several studies have demonstrated the important role of SREBP-1c, a major transcription factor involved in hepatic lipogenesis, which leads to increases in fatty acid synthesis as a result of the induction of FAS and ACC [29,30]. One study reported that the level of SREBP-1c showed positive correlation with the degree of hepatic steatosis in patients with NAFLD [31]. Results of the current study showed that the level of SREBP-1c was significantly higher in the liver of rats fed a HFD compared with that of ND rats. However, SBS effectively inhibited the raise of SREBP-1c expression. FAS catalyzes the late step in fatty acid biosynthesis, thus, it is believed to be a major determinant of maximal hepatic capacity for generation of fatty acids by de novo lipogenesis. According to these results, administration of SBS resulted in decreased HFD-induced high expression of ACC and FAS, and expression of SREBP-1c transcriptional targets FAS and ACC showed strong correlation with SREBP-1c expression, suggesting that suppression of ACC and expression of FAS may contribute to a reduction in lipid accumulation in fatty liver. Together, since SBS supplementation in HFD rats resulted in significantly reduced serum and hepatic triglyceride, and cholesterol concentrations, these results demonstrated that SBS induced down-regulation of lipogenesis-related genes in HFD-induced fatty liver. Of particular importance, PPARα is a ligand-activated transcription factor; its activation induces the mRNA expression of several genes involved in fatty acid oxidation to reduce the circulating lipid levels [32]. The current results showed significantly lower PPARα expression in the HFD group than in the ND group, and it was increased by BS supplementation. Findings of a recent report indicated that PPARα also modulates hepatic lipogenic gene expression, such as DGAT, ACC, and PGC-1α, which are closely related to fatty acid synthesis and oxidation in hepatic steatosis in HFD-fed animals [33]. DGAT is a microsomal enzyme that joins acyl-CoA to 1,2-diacylglycerol and thus constitutes the final step in TG biosynthesis [34]. Our results showed that DGAT2 expression in HFD rats was significantly increased, while DGAT2 mRNA expression showed a dramatic decrease in the SBS-treated group. Recent studies have suggested that overexpression of DGAT2 leads to increases in large cytosolic lipid droplets [34], and hepatic-specific overexpression of DGAT2 led to increased liver TG content [35]. Therefore, our results indicated that administration of SBS resulted in down-regulation of the levels of DGAT2 mRNA and reduction of DGAT2 expression in the HFD-SBS group inhibited accumulation of hepatic lipid droplets via a decrease of TG synthesis. In the current study, we also found that mRNA expression of PPARγ was significantly lower in the FHD-SBS group than in the HFD group, demonstrating that a SBS-supplemented diet induced a reduction of PPARγ expression in the HFD-induced steatosis liver. In addition, gene expression generally exhibits a negative relationship with the DNA methylation of CpG islands in the gene promoter region. Sookoian *et al.* [36] reported that an increase of PPAR-γ coactivator 1α methylation correlated with decreased mRNA expression in the liver and contributed to insulin resistance in NAFLD patients. In HFD-fed mice, the increased methylation of the *PPARγ* promoter accompanied by the decreased expression of *PPAR*γ mRNA in visceral adipose tissues was associated with the pathogenesis of metabolic syndrome [37]. Taken together, our results showed a significantly smaller liver lipid droplet area in the SBS group, accompanied by an increase in PPARα and a decrease in expression of SREBP-1c, FAS, ACC, and DGAT2.

Medicinal plants or many natural products exhibiting good antioxidant activity are associated with hepatoprotection potential. Bamboo leaves exhibited significant antioxidant activity against the 1,1-diphenyl-2-picrylhydrazyl (DPPH) radical and a cytoprotective effect against oxidative damage in liver cells [17]. In the current study, we found that SBS extracts contained high phenolic contents (430 mg gallic acid equivalent/g extract) and flavonoid contents (127.5 mg quercetin equivalent/g extract), respectively. When we analyze SBS extracts using with HPLC-DAD, in terms of phenolic acids, SBS extracts had phloroglucinol, 4-hydroxy benzhydrazide, garlic acid, vanillic acid, caffeic acid, syringic acid, chlorogenic acid, *p*-coumaric acid, *trans*-ferulic acid, sinapic acid, 2-amino-3,4-dimethyl-benzoic acid, protocatechuic acid, and coumarin. Among phenolic compounds, protocatechuic acid, coumarin, and *p*-coumaric acid were the main materials. In addition, SBS extracts also had many flavonoids, such as gallocatechin, epigallocatechin, catechin hydrate, epicatechin, epigallocatechin gallate, rutin hydrate, naringin, quercetin hydrate, myricetin, quercetin dehydrate, luteolin, and kaempferol. Our results also showed that SBS had an effective capacity of scavenging for DPPH, ABTS, and hydroxyl radicals and showed correlation with potent phenol and flavonoid contents, thus, suggesting its antioxidant potential. The current finding suggested that hepatoprotective effects and anti-steatosis functions of SBS in HFD-induced NAFLD may be due to the potent antioxidant properties and to the abundant presence of phenolic and flavonoid contents of SBS. 

## 5. Conclusions

In conclusion, these results indicated that SBS induced significant suppression of TG and cholesterol accumulation in the liver of rats fed a HFD. SBS possesses a repressive property on hepatic steatosis, which is associated with inhibition of SREBP1c, PPARγ, ACC, DGAT2, and FAS, and induction of PPARα, suggesting a potential application of SBS in treatment of HFD-induced NAFLD. 
